# Pancreatic Carcinoma Causing Schnitzler’s Metastasis

**DOI:** 10.7759/cureus.42465

**Published:** 2023-07-25

**Authors:** Laya B Giri, Julia Sunil, Norton Stephen, Oseen Shaikh, Gopal Balasubramanian

**Affiliations:** 1 Surgery, Jawaharlal Institute of Postgraduate Medical Education and Research, Puducherry, IND; 2 Pathology, Jawaharlal Institute of Postgraduate Medical Education and Research, Puducherry, IND

**Keywords:** intestinal obstruction, carcinoma stomach, carcinoma pancreas, rectal stenosis, schnitzler’s metastasis

## Abstract

Schnitzler’s metastasis occurs due to the deposition of the tumor cells in the submucosa of the rectum, leading to rectal stenosis. We present a 60-year-old female who presented with abdominal pain, distension, and vomiting. Abdominal examination showed a distended abdomen and palpable bowel loops, and per rectal examination showed rectal stenosis. Imaging studies suggest rectal stenosis with carcinoma of the pancreas head. The patient was diagnosed with Schnitzler’s metastasis with carcinoma of the pancreas head, which has not been reported in the literature. The patient underwent a diversion sigmoid colostomy and was planned for palliative chemotherapy after stenting the common bile duct.

## Introduction

Schnitzler’s metastasis (SM) is rare in diffuse gastric cancer. They occur due to peritoneal or hematogenous spread [1,2]. SM is characterized by narrowing of the rectum, causing stenosis and bowel obstruction. Patients may present with constipation or intestinal obstruction. Imaging studies like computed tomography (CT) and magnetic resonance imaging (MRI) suggest rectal stenosis with normal mucosa [3]. Management of such patients is usually chemotherapy unless they have an intestinal obstruction. The patient can undergo a diversion or formal rectal resection in this case. We report a 60-year-old female patient who presented with intestinal obstruction and was diagnosed with SM. The patient underwent diversion sigmoid colostomy and common bile duct (CBD) stenting and was planned for palliative chemotherapy.

## Case presentation

A 60-year-old female patient complained of abdominal pain and distention for 10 days. The patient also had a history of vomiting for three days. The patient’s neighbors noticed she had jaundice for two weeks before visiting the hospital. There was no history of fever, hematochezia, or hematemesis. On examination, she had a pulse rate of 90 beats per minute and a blood pressure of 90/60 mm Hg. There was jaundice and mild pitting pedal edema. There was no lymphadenopathy in the neck, axilla, or inguinal region.

Abdominal examination showed a distended abdomen. There was mild generalized tenderness but no guarding or rigidity. Bowel loops were palpable with visible peristalsis. There was no evidence of hepatomegaly or splenomegaly. Per rectal examination showed a severely stenosed rectum, unable to administer a finger across the stenosed rectum. There was no palpable growth within the rectum. Rectum was empty without any stool staining. Routine investigations showed hemoglobin of 8 g/dl and mild leucocytosis. The renal function test (RFT) was normal. The liver function test (LFT) showed increased total bilirubin of 16 mg/dL, direct bilirubin of 12 mg/dL, and alkaline phosphatase (ALP) of 495 IU/L. Tumor markers like carcinoembryonic antigen (CEA) were 2 ng/mL, and carbohydrate antigen 19-9 (CA19-9) was more than 200 U/mL.

**Figure 1:**
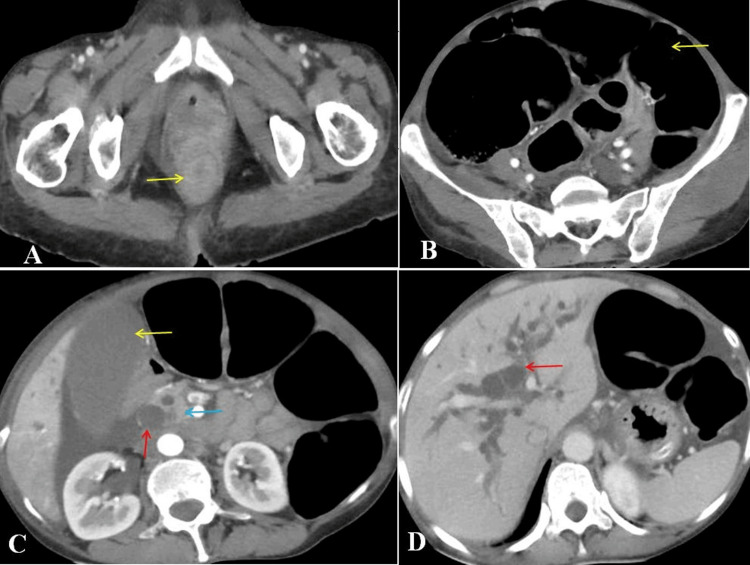
Compute tomography showing (A) circumferential thickening of the rectum (arrow) causing complete obstruction, (B) massively dilated large bowel loops (arrow), (C) dilated common bile duct (red arrow), distended gallbladder (yellow arrow), and pancreatic mass lesion (blue arrow), and (D) intrahepatic biliary radical dilatation (arrow) without any evidence of metastasis.

Contrast-enhanced CT showed a strictured rectum with complete narrowing, without any pelvic or abdominal deposits, liver metastasis, or lung metastasis. There was evidence of a tumor in the pancreatic head with dilated CBD and intrahepatic biliary radical dilatation (IHBRD). There was no evidence of metastatic deposits in the pelvis or the abdomen. There was evidence of dilated small and large bowel loops (Figures 1A-1D).

The patient underwent an emergency diversion sigmoid colostomy due to a fully developed intestinal obstruction. We did a per rectal examination intraoperatively and noted similar findings. We also did a per stomal examination from the distal loop of the sigmoid. There was no palpable growth in the rectum. The patient improved well postoperatively, and her symptoms subsided.

**Figure 2:**
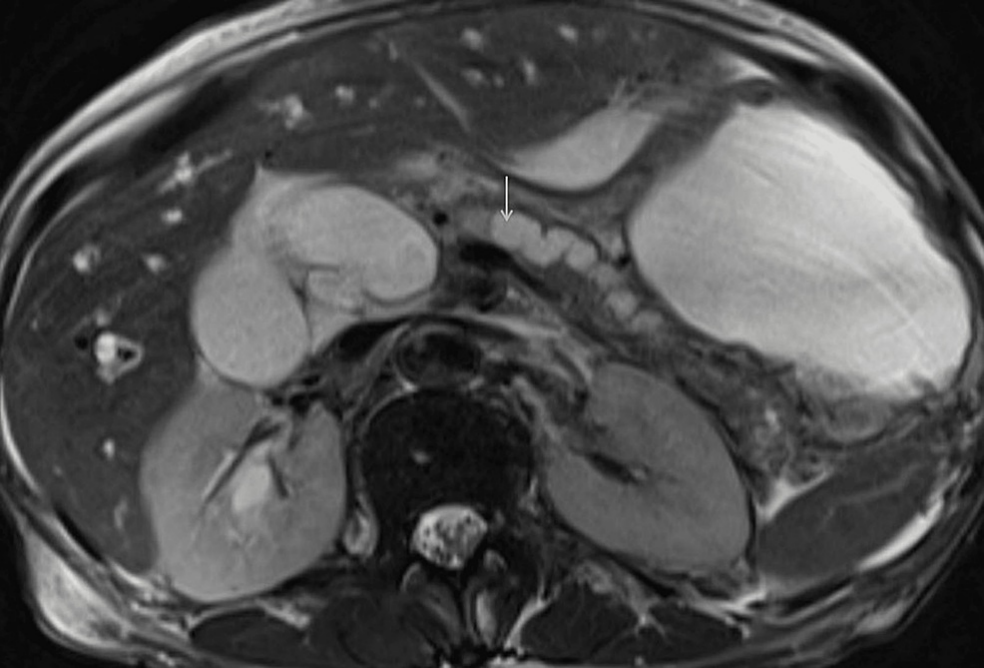
Magnetic resonance cholangiopancreatography (axial image) showing dilated common bile duct and pancreatic duct (arrow).

The patient underwent sigmoidoscopy and was found to have severe rectal stenosis, and the scope could not be negotiated beyond. She also underwent magnetic resonance cholangiopancreatography (MRCP). She was found to have an ill-defined hypointense T2 image and isointense T1 lesion measuring 1.5 cm x 2 cm in the pancreas head with mild homogenous enhancement and restricted diffusion. The pancreatic duct and CBD were dilated with abrupt tapering (Figure 2).

**Figure 3:**
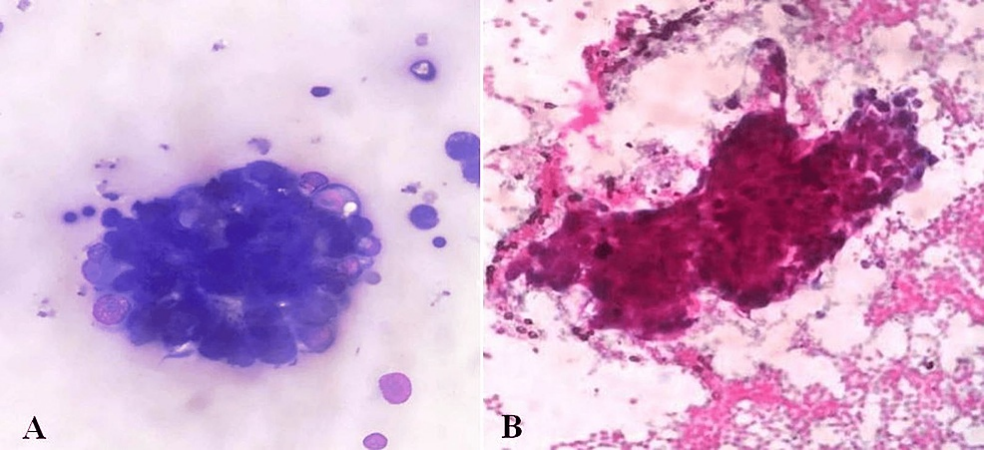
Cytological image showing tumor cells showing hyperchromatic pleomorphic nuclei with scant cytoplasm. (A) May Grunwald-Giemsa (MGG) stain and (B) Papanicolaou (PAP) stain.

Hence, she underwent endoscopic ultrasound (EUS) and fine needle aspiration (FNA) to diagnose the pancreatic head lesion. The stomach was normal, and there was no evidence of malignancy. EUS also showed the presence of a hypoechoic lesion in the pancreas head, suggesting malignancy. FNA from the lesion showed scattered tumor cells with hyperchromatic pleomorphic nuclei with scant cytoplasm (Figures 3A, 3B).

These cells were positive for cytokeratin (CK) 7 and CK 19, suggesting a pancreatic-origin adenocarcinoma. Considering all these findings, we diagnosed our patient with carcinoma of the pancreatic head with SM. After CBD stenting, supportive care and palliative chemotherapy were planned for the patient.

## Discussion

Metastasis to the rectum from gastrointestinal malignancies is uncommon. SM is a type of rectum metastasis that can be synchronous or metachronous. It is characterized by severe rectal stenosis with circumferential diffuse wall thickening and tumor-free rectal mucosa [1]. Only a few case reports are published in the SM literature [1,3-6]. All reported cases had gastric carcinoma as the underlying cause. Gastric carcinoma is divided into two types: the intestinal type and the diffuse type. The intestinal type of carcinoma is more commonly spread by lymphatic spread, whereas the diffuse type spreads through hematogenous spread [2]. The usual sites of metastasis from stomach cancer include Virchow's node, Krukenberg's tumor, and Blummer's shelf, which occur due to the peritoneal spread of the tumor. SM is a rare metastasis due to venous and lymphatic spread from poorly differentiated gastric carcinoma [3]. A similar spread mechanism can occur in a primary pancreatic malignancy. There are case reports in the literature where pancreatic malignancy had some unusual sites of metastasis, like the descending colon, sigmoid colon, skeletal muscle, skin, and subcutaneous tissues [7-10]. To our knowledge, there is no case report in the literature where pancreatic malignancy causes rectal stenosis, causing SM.

Most SM patients were females [11]. Patients with SM presented with abdominal pain, constipation, tenesmus, rectal discharge, and abdominal distention [1]. Similarly, patients with pancreatic malignancy with colonic and sigmoid metastasis can develop intestinal obstruction. When a patient is diagnosed with SM, they will have a stenosed rectum on their per-rectal examination. Our patient was also female, had pancreatic head malignancy with SM, and presented with intestinal obstruction.

X-ray abdomen may reveal features of intestinal obstruction in patients who have developed frank features of intestinal obstruction. However, the etiology of intestinal obstruction cannot be commented on. CT abdomen may show concentric stenosis of the rectum [1]. Few patients may develop an onion ring stenosis. Perirectal fat may show fibrosis but no infiltration of surrounding structures [3]. MRI has excellent soft tissue resolution; hence MRI is the preferred modality for evaluating rectal disorders [12]. However, in patients with a frank intestinal obstruction, the utility of MRI is doubtful as, in most places, MRI may not be available during emergency hours. Our patient underwent CT for intestinal obstruction and was diagnosed with severe rectal stenosis with pancreatic head malignancy.

Endoscopic evaluation of the rectum is the most effective way to assess mucosal lesions. In patients who develop SM, endoscopic evaluation may show normal mucosa with complete narrowing of the rectum lumen. Due to the stenosed rectum, it may not be possible to pass the scope beyond the rectal narrowing [1]. Our patient had a severely stenosed rectum, and the scope could not be negotiated.

Treatment of patients with SM depends on the clinical presentation of the patients. Those who have developed frank features of intestinal obstruction will need surgical management. Most patients will need a proximal colon diversion [4]. There are case reports in the literature where patients with SM have undergone ultra-low anterior resection, Hartmann’s procedure, and self-expanding metal stents [1,4,13]. Few patients, who did not have intestinal obstruction features, received only chemotherapy [6]. Our patient underwent a diversion sigmoid colostomy and later CBD stenting and palliative chemotherapy.

## Conclusions

SM is considered the rarest type of metastasis associated with diffuse gastric carcinoma. SM occurs through the hematogenous or lymphatic spread. Considering that no pre-existing literature describes SM associated with pancreatic malignancy, our case would be the first to describe such a scenario. Tumor cells are usually deposited in the rectum submucosa. Patients may present with symptoms of intestinal obstruction. MRI may be more beneficial for diagnosis. SM treatment can be tailored according to the clinical presentation of the patient.

## References

[1] Derici ZS, Sokmen S (2016). Gastric carcinoma presenting with severe rectal stenosis: ‘schnitzler’s metastasis’: case report and review of the literature. Eur Surg.

[2] van der Woude CJ, Kleibeuker JH, Tiebosch AT, Homan M, Beuving A, Jansen PL, Moshage H (2003). Diffuse and intestinal type gastric carcinomas differ in their expression of apoptosis related proteins. J Clin Pathol.

[3] Olano C, De Simone F, Gonzalez F, Gonzalez N, Tchekmedyian A, Pose A, Iade B (2009). Stomach cancer presenting with rectal stenosis: Schnitzler's metastasis. Gastrointest Endosc.

[4] Rausei S, Frattini F, Dionigi G, Boni L, Rovera F, Diurni M (2010). Unusual rectal stenosis. J Surg Oncol.

[5] Lim SW, Huh JW, Kim YJ, Kim HR (2011). Laparoscopic low anterior resection for hematogenous rectal metastasis from gastric adenocarcinoma: a case report. World J Surg Oncol.

[6] Yamamoto M, Matsuyama A, Kameyama T (2008). A case of advanced gastric cancer with Schnitzler's metastases effectively treated by the combination of paclitaxel and S-1 (Article in Japanese). Gan To Kagaku Ryoho.

[7] Park DY, Krishnamurthi S, Chahal P, Downs-Kelly E, Morris-Stiff G (2019). Pancreatic metastases to the colon: an unusual cause of colonic obstruction. BMJ Case Rep.

[8] Borad MJ, Saadati H, Lakshmipathy A (2009). Skeletal metastases in pancreatic cancer: a retrospective study and review of the literature. Yale J Biol Med.

[9] Kahl R, George K, Patel K, Stawick L (2019). Pancreatic adenocarcinoma with rare sigmoid colon metastasis. ACG Case Rep J.

[10] Shi Y, Li SS, Liu DY, Yu Y (2020). Cutaneous metastases of pancreatic carcinoma to the labia majora: a case report and review of literature. World J Gastrointest Oncol.

[11] Dogan S, Demirbas S, Samadov E, Ozis SE, Uslu HY (2019). Rectal resection for schnitzler’s metastasis in a patient presenting with severe rectal stenosis: case report and review of the literature. Eur Res J.

[12] Balcı S, Onur MR, Karaosmanoğlu AD, Karçaaltıncaba M, Akata D, Konan A, Özmen MN (2019). MRI evaluation of anal and perianal diseases. Diagn Interv Radiol.

[13] Okugawa T, Oshima T, Ikeo K (2013). Successful self-expandable metallic stent placement for a case of distal rectal stenosis due to gastric cancer metastasis. Case Rep Gastroenterol.

